# Sensitivity comparison for the *Leishmania* spp. detection in different canine tissues using PCR-HRM

**DOI:** 10.1590/0037-8682-0069-2022

**Published:** 2022-12-16

**Authors:** Ana Fidelina Gómez Garay, Stefania Fraenkel, Jorge Javier Alfonso Ruiz Diaz, Oscar Daniel Salvioni Recalde, María Celeste Vega Gómez, Jorge Arístides Miret Riquelme, Paola Verónica Arze, Gloria Natalia Ramírez Centurión, Milena Britos, Miriam Rolón

**Affiliations:** 1 Centro para el Desarrollo de la Investigación Científica, Asunción, Central, Paraguay.; 2 Ministerio de Salud Pública y Bienestar Social, Programa Nacional de Control de Zoonosis y Centro Antirrábico Nacional, San Lorenzo, Central, Paraguay.; 3 Universidad Nacional de Asunción, Instituto de Investigaciones en Ciencias de la Salud, Departamento de Medicina Tropical, San Lorenzo, Central, Paraguay.

**Keywords:** Hsp70, Stray dogs, rk39, PCR-HRM, Paraguay

## Abstract

**Background:**

Leishmaniasis is a vector-borne disease caused by a parasite protozoon from the genus *Leishmania*. Among the molecular techniques applied for detecting these parasites, real-time PCR with High Resolution Melting (PCR-HRM) proved advantageous since it simultaneously determines both the presence and species of the pathogen in one step, through amplification and later analysis of curves generated by melting temperature.

**Methods:**

Based on this molecular technique, the goal of this study was to estimate the PCR-HRM sensitivity for *Leishmania* spp. detection in different canine tissues by evaluating biological samples obtained from popliteal, submandibular, and pre-scapular lymph nodes, from bone marrow and ear pinnae of 28 stray dogs captured in the metropolitan area of Asunción (Paraguay).

**Results:**

The rk39 immunochromatographic test showed that 25/28 tested dogs (89%) presented antibodies against *L. infantum*. In 20/25 dogs that tested positive for rk39 (80%), it was possible to detect *Leishmania* spp. by PCR-HRM and determine that the species corresponded entirely to *L. infantum*. Regarding the analysis of different tissues, the parasite was detected in all popliteal lymph node samples, followed by high detection in submandibular (at 95%) and pre-scapular lymph nodes (at 90%), bone marrow (at 85%), and ear pinnae (at 85%).

**Conclusions:**

This study demonstrated that the use of real-time PCR-HRM using the molecular marker hsp70 was a highly sensitive method for simultaneously detecting and identifying *Leishmania* species in different tissues taken from infected dogs. In addition, the usefulness of ear pinnae as easily accessible tissue for molecular diagnosis was emphasized.

## INTRODUCTION

According to World Health Organization (WHO), leishmaniasis is considered a neglected tropical disease (NTD) which is widely distributed in tropical and sub-tropical areas, primarily affecting less developed and developing countries^1^ ^,^ ^2^. It is caused by unicellular flagellate parasites of the genus *Leishmania* spp. that circulate between the vector and the hosts to carry out their life cycle, multiplying as free promastigotes in the intestinal lumen of the vector while proliferating as obligatory intracellular amastigotes in the macrophages of the hosts^3^.

The disease is prevalent in 92 countries around the world. Its main clinical manifestations in humans include epithelial injuries, characteristic of Cutaneous Leishmaniasis (CL), and lesions in the liver and spleen^2^ ^,^ ^4^ in the case of Visceral Leishmaniasis (VL). In addition, there is Mucocutaneous Leishmaniasis (ML) infection with primarily oral and nasal injuries that incapacitate the carrier. Although the presence of the parasite is global, most cases are reported in the continents of Africa, Asia, and South America^4^ ^,^ ^5^. In Paraguay, 48 cases of leishmaniasis in humans were diagnosed between the years 2018 and 2021, of which 20 were VL and 28 of the remaining cases included CL and ML^6^.

In the context of the presence of leishmaniasis in urban areas, domestic dogs (*Canis familiaris*) are of great epidemiological importance since they constitute the most important reservoir of the parasite. Moreover, dogs may normally present a high parasitic load which naturally constitutes a health threat to the humans that live with them^7^ ^,^ ^8^ ^,^ ^9^. In this sense, an increase in the frequency of cases of Canine Visceral Leishmaniasis (CVL) has been observed as a phenomenon that preceded an increase in human cases^10^ ^,^ ^11^. Studies aimed at investigating the prevalence of antibodies against *Leishmania* in dogs signal that in high endemic areas, the presence of antibodies could reach between 50 and 75%^12^. Previous serological studies of stray dogs in the metropolitan area of Asunción, captured within a 500-meter radius area around a human case detection point (n=42,000), showed a prevalence of the VL case, with antibody count at 69% according to the rk39 test results^13^.

In relation to the diagnosis of CL, the sensitivity of the technique used to detect the presence of the parasite in a sample depends on the parasitic load and the resolution of the applied technique. It has been observed that any one of them could lead to divergent results in some cases^14^. Furthermore, diverse studies suggest the necessity to adopt one or a variety of criteria to decrease the occurrence of false negative or false positive results in the analyzed samples^15^.

Conventionally, the presence of *Leishmania* spp. genus parasites can be determined by the parallel application of various techniques, such as the direct observation of the parasite in blood smear, culture, and isolation of samples of different tissues; use of serological assays, such as the Indirect Fluorescent Antibody Technique (IFAT); use of direct agglutination; application of rk39 rapid test; and others^16^. It is noteworthy that although these are routinely applied techniques, they have limitations and, in most cases, these procedures do not detect the presence of the parasite in asymptomatic individuals^17^.

In recent years, a rapid evolution of the molecular diagnostic techniques by Polymerase Chain Reaction (PCR) and its different variants, as well as the utilization of different molecular markers, contributed considerably to the detection of the parasite in an easy and sensitive manner^18^. Among the molecular detection methods, High Resolution Melting Analysis (HRM), post-PCR, highlights its capacity to detect the presence of the parasite and identify the species, all in one step through curves generated by the temperature of fusion, which are related to the G+C content of the amplified fragment^19^ ^,^ ^20^.

Thus, in this manner, the aim of this study was to compare the detection sensitivity of *Leishmania* spp. in different tissues while simultaneously determining the species of the infectious parasite by using the PCR-HRM technique for samples collected from stray dogs that were captured in the city of Asunción and previously subjected to the rapid immunological rk39 test by Ministry of Public Health and Social Welfare protocol.

## METHODS

### Stray Dogs

The study population consisted of 28 stray dogs--14 females and 14 males of different breed and ages--captured in the metropolitan area of Asunción (Table 1). The stray dogs were sampled between April and June of 2013 and maintained in the premises of the National Anti-rabies Center (*CAN*, in its Spanish acronym), an agency dependent of the Ministry of Public Health and Social Welfare (*MSPyBS*, in its Spanish acronym) and which is part of the National Program for Zoonotic Diseases, in Paraguay. The study was conducted on stray dogs captured within a 500-meter radius circle around a point of human case occurrence of VL and subsequently tested by rk39 strip by MSPyBS guidelines.

**TABLE 1:** Clinical characteristics, and immunological and molecular diagnosis of leishmaniasis in dogs studied in the metropolitan area of Asunción.

Specimen	Breed	Age (Years)	Gender	Clinical signs	Results of rk39	Results of qPCR hsp70 by tissue				
						BM	EP	PN	PSN	SMN
1	Mixed Terrier	3	M	Conjunctivitis, hepato and splenomegaly	+	+	+	+	+	+
2	Mixed	3	F	Dermatitis, ganglionar hypertrophy, long nails, pinna injury, hepato and splenomegaly	+	+	+	+	+	+
3	Mixed	10	M	Long nails, hepato and splenomegaly	+	-	-	**	-	-
4	Mixed	6	F	Dry hair, node hypertrophy, long nails, seborrhea, weight loss, hepato and splenomegaly	+	+	+	+	+	+
5	Mixed	5	F	Seborrhea, conjunctivitis, scabs on the skin, hypertropia ganglionar, hepato and splenomegaly	+	+	+	+	+	+
6	Mixed	5	F	Long nails, pinna injury, node hypertrophy, eye ulcer, anemia, hepato and splenomegaly	+	+	+	+	+	+
7	Poodle	3	M	Cachexia, long nails, anemia, hepato and splenomegaly	+	+	+	+	+	+
8	Mixed	7	M	Cachexia, long nails, dry hair, hair loss, cutaneous ulcers, node hypertrophy, seborrhea, hepato and splenomegaly	+	+	+	+	+	**
9	Mixed	10	F	Long nails, dry hair, hair loss, cutaneous ulcers, node hypertrophy, seborrhea, conjunctivitis, plantar hyperqueratosis, weight loss, hepato and splenomegaly	+	-	-	-	-	-
10	Mixed	3	M	Long nails, dry hair, nasal and plantar hyperqueratosis, seborrhea, conjunctivitis, nasal ulcer, hepato and splenomegaly	+	-	-	-	-	-
11	Poodle	>1	F	Alopecia, weight loss, cutaneous ulcers, seborrhea, hepato and splenomegaly	+	+	-	+	+	+
12	Mixed	7	F	Weight loss, long nails, hair loss, conjunctivitis, plantar hyperkeratosis, hepato and splenomegaly	+	-	-	-	-	-
13	Poodle	3	M	Weight loss, long nails, seborrhea, ear ulcer, hepato and splenomegaly	+	+	+	+	+	+
14	Poodle	6	F	Weight loss, long nails, conjunctivitis, node hypertrophy, seborrhea, hepato and splenomegaly	+	+	+	+	+	+
15	Mixed	5	M	Asymptomatic	+	+	+	+	+	+
16	Mixed	4	F	Long nails, cachexia, dry hair, anemia, hepato and splenomegaly	+	+	+	+	+	+
17	Mixed	2	M	Long nails, blepharitis, conjunctivitis, plantar hyperkeratosis, dry hair, node hypertrophy, seborrhea	+	+	+	+	+	+
18	Mixed	1	M	Plantar hyperkeratosis, hepato and splenomegaly	+	-	-	+	-	+
19	Mixed	5	M	Long nails, cutaneous ulcers, conjunctivitis, anemia, seborrhea, weight loss, node hypertrophy, conjunctivitis	+	+	+	+	+	+
20	Mixed	2	M	Seborrhea, conjunctivitis, weight loss, long nails, lagana, scites, hepato and splenomegaly	+	-	-	-	-	-
21	Mixed	7	F	Cachexia, hyperkeratosis, dry hair, ear ulcer, alopecia	+	+	+	+	+	+
22	Mixed	7	F	Hyperkeratosis, cutaneous ulcers, seborrhea, weight loss, long nails, conjunctivitis, node hypertrophy	+	-	+	+	+	+
23	Mixed	7	F	Dry hair, long nails, alopecia, dermatitis	+	-	+	-	-	-
24	Pitbull	>1	F	Eczema, weight loss, cutaneous ulcers, node hypertrophy, edema de members, plantar and nasal hyperkeratosis	+	+	+	+	+	+
25	Pitbull	2	M	Cachexia, plantar hyperkeratosis, seborrhea, dry hair, cutaneous ulcers	+	+	-	+	+	+
26	Mixed	3	M	Asymptomatic	-	-	-	-	-	-
27	Mixed	4	F	Asymptomatic	-	-	-	-	-	-
28	Mixed	2	M	Asymptomatic	-	-	-	-	-	-

**BM:** bone marrow; **EP:** ear pinna; **PN:** popliteal lymph node; **PSN:** pre-scapular lymph node; **SMN:** submandibular lymph node. (**) Sample not obtained. **F:** female; **M:** male.

### Tissue samples and clinical signs

Five different tissue samples were taken from each animal: from the popliteal node (PN), submandibular node (SMN) and pre-scapular lymph nodes (PSN), the bone marrow (BM), and the ear pinnae (EP). The PN, SMN, PSN, and BM samples were taken by puncture and aspiration with a 1 mL syringe, while the EP samples were obtained by deep scraping with a scalpel blade.

**FIGURE 1:**
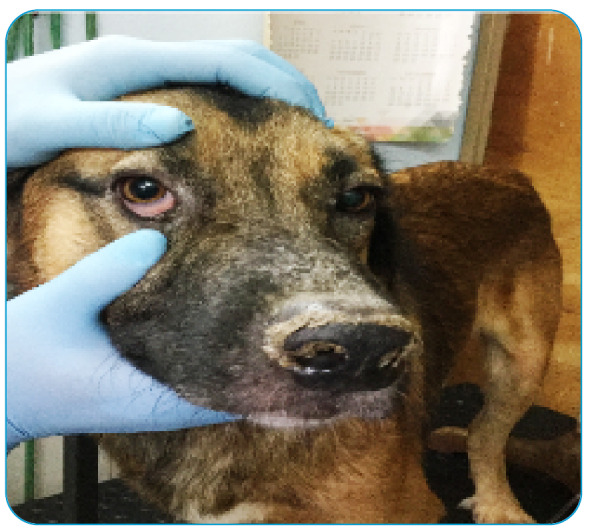
*Canis familiaris* specimen with dermal injuries and ulcers typical of leishmaniasis.

The gender, breed, characteristics, and clinical signs of the disease were observed and registered by professional veterinarians from CAN and the details are presented in Table 1 and Figure 1. 

### Serological test rk39

Serological tests were performed by CAN using the lateral flow chromatography using the rk39 strip, following the instructions by the manufacturer (https://diagnostics.be/). The samples of serum previously separated by centrifugation were placed on top of the test strip and incubated with a buffer. Visual readings for identification were performed after 10 min; those readings that showed a definite band pattern for this assay were considered as a positive result^21^.

### DNA extraction and quantification of *L. (L.) infantum* strain

*L.* (L.) *infantum* promastigotes (MCAN/ES/92/BCN83) were maintained in culture at 26 °C using Schneider's Insect Medium (Sigma-Aldrich®) supplemented with 10% inactivated fetal bovine serum (FBS; Sigma-Aldrich®). Parasite DNA extraction was performed in its stationary phase equivalent to 1×10^6^ parasites/mL using the commercial GeneJET Genomic DNA Purification Kit (#K0722 Thermo Scientific®), following the manufacturer's instructions. Subsequently, successive dilutions with a 1:10 factor (26 ng/µL *L.* (L.) *infantum* DNA) were performed to evaluate C_t_ values by absolute quantitative qPCR.

### DNA extraction and purification of tissue samples

From 26 dogs, the five tissue samples required for this study were obtained; in 2 dogs, only four of the five tissue samples were collected. In total, 138 samples were gathered. DNA of each tissue was extracted and purified in CEDIC using the Gene JET Genomic DNA Purification Kit® (#K0722; Thermo Scientific, Waltham, MA), following the instructions of the manufacturer. At the end of each extraction, the degree of purity of the genetic material was evaluated using a spectrophotometer (DS-11FX + DeNovix®, Wilmington, DE). Good laboratory practices were used to avoid DNA cross contamination. 

### DNA amplification and quantification of *Leishmania* spp.

To confirm the presence and absolute quantification *Leishmania* spp., a fragment of 144bp of the heat shock protein 70 gen (hsp70) was amplified with real-time PCR, using the primers Fhsp70F2 5ʹ-GGAGAACTACGCGTACTCGATGAAG-3ʹ and Rhsp70C 5ʹ-TCCTTCGACGCCTCCTGGTTG-3ʹ, as described by Zampieri et al.^22^. The qPCR was performed in a final volume of 20 μL, using 10 μL of the HRM PCR MasterMix® (QIAGEN, Germantown, MD), with a DNA final concentration ~30 ng/μL ,and both primers with a final concentration of 0.5 µM.

The qPCR was executed in the Rotor-Gene 6000® (QIAGEN) thermocycler, and the cycling conditions were: initial denaturation at 95 ºC for 10 min, followed by 40 cycles of denaturation at 95 ºC for 10 s, annealing at 60 ºC for 30 s, and extension at 72 ºC for 10 s.

For quantitative assays, a standard curve was performed in three successive and independent qPCR assays, using 3 μL of *L* (L.) *infantum* DNA (MCAN/ES/92/BCN83). Using the dilutions and PCR conditions mentioned above, in each qPCR, water was used as reaction control (NCT). The efficiency of all assays was analyzed using the LinRegPCR software (version 2021.2) and Excel (Office® 2016).

Finally, a comparison of the parasite load was carried out to determine significant differences in the DNA concentration of *L. infantum* in the different tissues. Data were submitted to analysis of variance (ANOVA) followed by the Tukey post-test with a significance level of p<0.05. Statistical analyses were performed using Graph Pad Prism version 6.0 software.

### Identification of *Leishmania* spp.

To determine the *Leishmania* species, the HRM analysis of the amplicon dissociation was performed immediately after the conclusion of real-time PCR. The melting range was established between 80 ºC and 90 ºC, with a slope of 0.1 °C/s. The HRM curve analysis was carried out with the Rotor Gene 6000 software version 2.1.0 (QIAGEN). DNA from the reference strain of *L.* (L.) *infantum* (MCAN/ES/92/BCN83) was used as a positive control and the *Leishmania* species present in the analyzed tissues were identified by comparing the melting profiles with that of the reference strain.

## RESULTS

Of the 28 dogs selected for this study, 25 (89%) were determined as positive for *Leishmania* spp. according to the rk39 strip test. There were also three asymptomatic and negative dogs for this immunological test which were used as negative control for the molecular assays (Table 1).

The clinical evaluation of the 25 positive animals by rk39 revealed signs of the disease, such as hepatosplenomegaly (68%), long nails (56%), ulcers and seborrhea (44%), conjunctivitis and weight loss (40%), dry hair and hyperkeratosis (32%), cachexia (20%), anemia (16%), hair loss (0,12%), and dermatitis and scabs on the skin (0,04%); meanwhile, the remaining 4% did not present any signs of the disease (Table 1).

**FIGURE 2:**
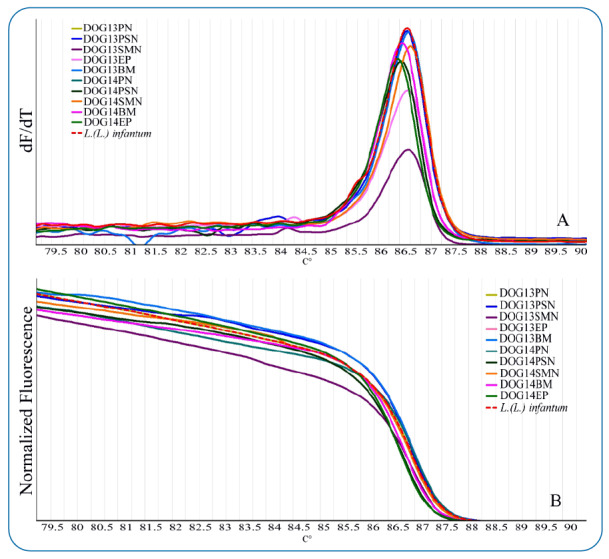
**(A)** Tm values obtained with the high-resolution melt (HRM) assay for positive control (*L.* (L.) *infantum*) and canine tissue samples. **(B)** Normalized curve of the heat shock protein (hsp70) amplicon obtained by HRM.

**SUPPLEMENTARY FIGURE 1:**
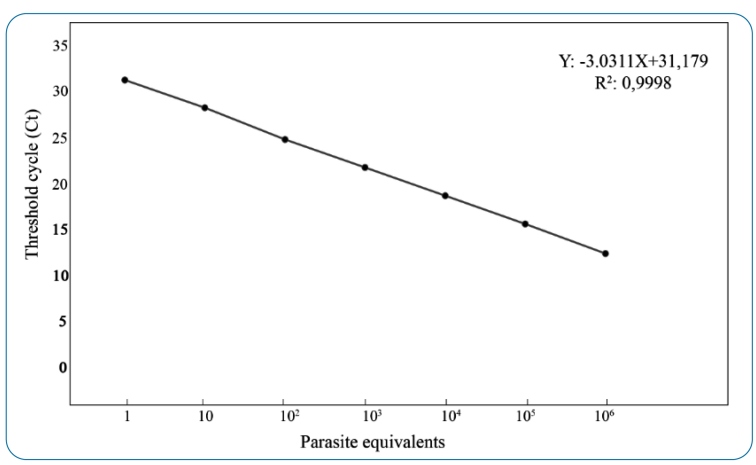
Standard curve target hsp70: threshold cycle values and equivalent parasite DNA by reaction.

The results of the standard curve provide the C_t_ (Threshold Cycle) values for each equivalent parasite concentration which were subsequently analyzed with the linear regression formula ( Supplementary Figure 1). Molecular results obtained by real-time qPCR of the tissues of the 28 dogs included in this study confirmed that 20 of the 25 positive dogs, according to rk39 results (80% of the population), were also positive for *Leishmania* spp. in one or more than one tissues (Table 1), while the results were negative in the three dogs that were included as negative control (Table 1). The HRM analysis revealed the presence of *L. infantum* in all positive tissues (Figure 2). 

In 15/20 molecularly positive dogs (75%), it was possible to detect the presence of the parasite from the five analyzed tissues including the estimated parasite load by absolute quantification (Table 1,  Supplementary Table 1). 

Evaluating the sensitivity of the molecular technique in the positive specimens, divided by the type of analyzed tissues, the parasites were detected in 100% of the samples of the PN, followed by high detection in the SMN (95%), PSN (90%), BM (85%), and EP (85%) (Table 2). In total, the presence of *L. infantum* was detected in 89/98 molecular positive tissues analyzed (91% of the population) (Table 2).

**TABLE 2:** Percentage of detection sensitivity of *L. infantum* by PCR-HRM in different tissues obtained from 20 positive dogs.

**PCR-HRM for *L. infantum* detection**		
Tissue	Positive tissue / Analyzed tissue (N)	Sensitivity (%)
Popliteal lymph node (PN)	19/19*	100
Sub mandibular lymph node (SMN)	18/19*	95
Pre-scapular lymph node (PSN)	18/20	90
Bone marrow (BM)	17/20	85
Ear pinna (EP)	17/20	85
**Total**	**89/98**	**91%**

*One sample of this tissue not obtained.

**FIGURE 3:**
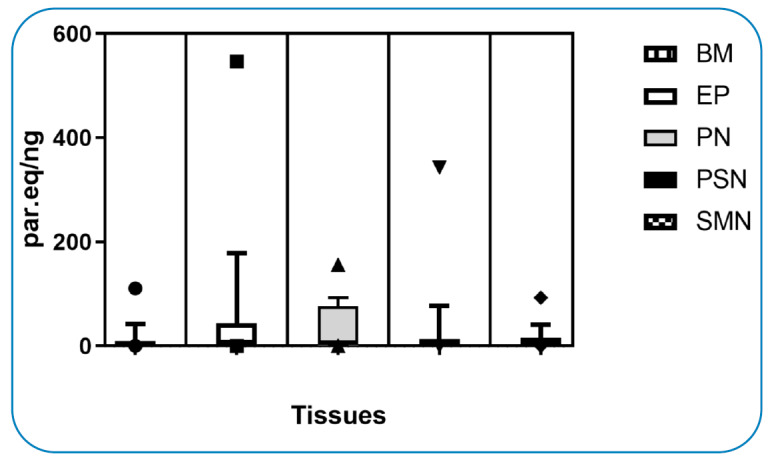
Comparative equivalent parasite DNA in tissues using ANOVA.

In addition to analyzing the parasitic load by qPCR in each tissue evaluated, there was no significant difference (p<0.05) when comparing the different tissues (Figure 3). However, when comparing parasitic loads between individuals, the animal that presented the highest parasitic load in the ear pinna corresponded to number 15, which did not present symptoms compatible with the illness (Table 1,  Supplementary Table 1).

## DISCUSSION

The genotypic detection and identification of parasites originating from vectors as well as vertebrate hosts fulfil an important part in the monitoring of zoonotic diseases. In this sense, domestic dogs are the main hosts of *L.* (L.) *infantum*, and accordingly have a fundamental part in the transmission cycle of leishmaniasis^23^. Thus, as a rapid diagnostic is fundamental to direct the treatment in human cases, the identification of reservoirs is just as essential^24^.

This study aimed to evaluate the applicability of the real-time PCR-HRM technique to simultaneously determine the presence and species of *Leishmania* spp., as well as the technique`s sensitivity in the different tissues obtained from stray dogs that had been naturally infected and captured in the metropolitan area of Asunción. Diverse methods exist that are can be used to detect *Leishmania* spp. in dogs, from a general method based on the detection of antibodies, often used with a limited efficiency, to the most sensitive and specific method such as the PCR based on samples obtained from distinct types of tissues^25^. Although the detection by direct observation of the parasites in tissues is still considered as the “golden standard” method, its applicability diminishes in cases where the parasitical load in the analyzed tissues is low. For this exact reason, in the last few years detection of the parasite by molecular methods was routinely put to the test, which allowed the development of different techniques^18^. Among these procedures, the one established by Zampieri and collaborators^22^ stands out, who, in 2016, reported the use of the PCR-HRM technique to differentiate *Leishmania* species. The fundamental principle of this technique is the detection of variations in the nucleotide sequence, which generates a pattern of characteristic curves that can be observed in real time^26^.

In Paraguay, the molecular detection of *Leishmania* spp. in samples of different sources has been traditionally performed using conventional PCR with restriction enzyme digestion^27^. Nonetheless, many recent local studies were focused on the application of the real-time technique for this same purpose^28^ ^,^ ^29^; in particular, scientists have started to use an innovative variation of the qPCR-HRM method, using the hsp70 marker for the detection and identification of *Leishmania* spp. species from human samples^30^.

The high specificity and sensitivity in the molecular detection of *Leishmania* spp. in samples of BM aspirate, nodes, and blood obtained from infected dogs have been previously reported^31^ ^,^ ^23^. In the context of this study, the obtained results showed that the samples extracted from distinct types of nodes showed higher levels of sensitivity, as previously described^32^ ^,^ ^33^, in comparison to the BM and EP samples. Although the literature mentions that by using the same technique, the bone marrow samples present similar sensitivity values compared to the lymph nodes, the lower sensitivity values observed in different samples in this study could have been due to the low parasitic load in the tissues or associated issues with the obtainment or processing of the samples^34^.

Regarding the sensitivity of the immunochromatography techniques used for diagnosis, the literature mentions that the rapid reveal of the strips is based on the detection of the recombinant rk39 antigen, which can detect the presence of parasites of the *L. donovani* complex. Additionally, studies showed that the main advantage of this technique is the combination of its efficiency and ease of use, since extensive training or a very sophisticated equipment is not needed for its interpretation^35^. The results of the rapid test compared to the ones of the real-time PCR obtained in this study revealed a confidence of 80%, similar to the values reported by other authors^25^ ^,^ ^36^. Nevertheless, the sensitivity reported by means of this technique in other studies is lesser if compared to the molecular methods, being dependent of the infection time and the parasitic load^37^. In this study the sensitivity of the previously mentioned immunochromatography test is greater when compared to the PCR-HRM results, since five dogs that had been diagnosed as positive by the rk39 test resulted negative in all the tissues according to the molecular analysis, which could have resulted in false positives. In addition, the inability to discriminate between immunity and actual infectiousness suggests that a combination with other non-immunological based tests will be required for highly sensitive/specific diagnosis in order to target control measures in individual reservoirs from a public health perspective, as for individual management from an animal health perspective^38^.

Considering the regions where there is a prevalence of leishmaniasis, combined with the socioeconomic characteristics that lead to it being considered as a neglected disease, the combination of two or more diagnostic methods is an alternative that should be considered. Another point to take into account is that urban dogs could be infected not only by *L. infantum* but also by *L. amazonensis* or *L. braziliensis*, as has already been described in different areas of Brazil^39^ ^,^ ^40^ ^,^ ^41^ ^,^ ^42^; therefore, the diagnosis of leishmaniasis in dogs should be confirmed using PCR based methods or by the isolation and subsequent characterization of the parasite using reference isoenzymatic methods^43^. As observed in this study, besides the election of the best detection method, the selection of the tissue to be analyzed is a relevant factor to be considered with the purpose of minimizing the stress caused to the animal when taking the sample.

An interesting finding aroused in this study is the high sensitivity of the epithelial tissue area for parasite detection, where a deep cutaneous scraping of the ear atrial margin of the dogs was performed in search of the *Leishmania* DNA. It should be noted that the detection of the parasite was performed in samples of healthy atrial epithelium, taken from areas without open wounds produced by the infection of *Leishmania* spp., thereby reproducing results presented by other authors^44^ ^,^ ^45^. The sensitivity of these tissues was 85%, similar to that observed in bone marrow samples, and coinciding with the results obtained by other authors^46^ ^,^ ^47^ ^,^ ^48^. In this way, considering the good sensitivity and less invasiveness of this technique when taking the sample, also taking into account that no significant differences were observed comparing the parasitic load in this tissue in relation to the other tissues, both the ear pinnae and the lymph nodes constitute a viable alternative for use in the diagnosis of Canine Leishmaniasis. However, future studies are necessary to determine the parasitic load in ear pinnae tissue and the detection limit for the molecular technique used in this study, under the objective of guaranteeing the least possible disturbance to the animal.

## SUPPLEMENTARY MATERIAL

**SUPPLEMENTARY TABLE 1:** Comparative equivalent parasite DNA value and ct by tissue.

BM				EP				PN		
Code	ct	*par.eq/ng		Code	ct	*par.eq/ng		Code	ct	*par.eq/ng
**1BM**	33.227	0.211		**1EP**	28.939	5.483		**2PN**	27.381	17.902
**2BM**	26.974	24.393		**2EP**	29.853	2.739		**4PN**	32.055	0.514
**4BM**	36.394	0.019		**4EP**	30.421	1.778		**5PN**	31.524	0.769
**5BM**	35.972	1.891		**5EP**	29.663	3.163		**6PN**	24.535	155.615
**6BM**	35.262	0.045		**6EP**	25.970	52.306		**7PN**	27.162	21.140
**7BM**	32.293	0.429		**7EP**	28.224	9.442		**8PN**	25.325	85.348
**8BM**	34.189	0.102		**8EP**	25.301	86.965		**11PN**	25.543	72.324
**11BM**	30.770	1.364		**13EP**	26.518	34.504		**13PN**	29.888	2.666
**13BM**	34.422	0.085		**14EP**	28.564	7.289		**14PN**	28.528	7.494
**14BM**	27.756	13.470		**15EP**	22.881	546.598		**15PN**	29.657	3.178
**15BM**	28.532	7.468		**16EP**	25.858	56.945		**16PN**	25.426	79.087
**16BM**	24.982	110.749		**17EP**	30.961	1.180		**17PN**	34.217	0.099
17BM	30.885	1.250		**19EP**	27.677	14.299		**19PN**	28.385	8.352
**19BM**	33.495	0.172		**21EP**	34.642	0.072		**21PN**	25.479	75.956
**21BM**	31.497	0.785		**22EP**	29.935	2.572		**22PN**	30.496	1.680
24BM	34.711	0.068		**23EP**	32.051	0.516		**24PN**	30.478	1.704
**25BM**	33.137	0.226		**24EP**	29.089	4.892		**25PN**	34.033	0.114
								**18PN**	25.378	81.991
**PSN**				**SMN**						
**Code**	**ct**	***par.eq/ng**		**Code**	ct	***par.eq/ng**				
**1PSN**	33.167	0.221		**1SMN**	33.302	0.199				
**2PSN**	27.763	13.393		**2SMN**	28.466	7.851				
**4PSN**	30.406	1.799		**4SMN**	30.062	2.337				
**5PSN**	32.870	0.277		**5SMN**	32.614	0.336				
**6PSN**	28.836	5.928		**6SMN**	27.560	15.631				
**7PSN**	30.234	2.051		**7SMN**	28.695	6.600				
**8PSN**	26.108	47.104		**11SMN**	33.448	0.178				
**11PSN**	28.669	6.732		**13SMN**	36.551	0.017				
**13PSN**	27.121	21.823		**14SMN**	29.971	2.503				
**14PSN**	32.095	0.499		**15SMN**	29.410	3.833				
**15PSN**	27.669	14.387		**16SMN**	25.215	92.827				
**16PSN**	23.494	343.183		**17SMN**	37.165	0.011				
**17PSN**	36.113	0.024		**19SMN**	27.581	15.388				
**19PSN**	27.855	12.494		**21SMN**	30.765	1.370				
**21PSN**	29.390	3.894		**22SMN**	31.711	0.667				
**22PSN**	33.212	0.213		**24SMN**	28.704	6.554				
**24PSN**	32.515	0.362		**25SMN**	37.274	34.711				
**25PSN**	33.232	0.210		**18SMN**	26.677	30.564				
